# Supplementary material for: Cryptosporidium varanii Infection in Captive Leopard Gecko (Eublepharis macularius) and Its Association with Wasting Syndrome in Thailand
Source: Animals (Basel). 2025 Dec 22;16(1):33. doi: 10.3390/ani16010033 (PMC12784683; doi:10.3390/ani16010033)
Supplement: Supplementary file 1 [file animals-16-00033-s001.zip › Supplement file Table S3.pdf]

Table S3 Nucelotide sequences of ssu-rRNA gene of *Cryptosporidium varanii* from this study.

Accession No.	Name	Sequence (5'-3')
PX496097	Cryptosporidium varanii LG16 Leopard gecko Chiang Mai Thailand	ATTATTTGGTGATTCATAATAACTTTACGGATCACAATTATTTGTGACATATCATTCAAGTTTCTGACCTATCAGCTTTAGACGGTAGGGTATTGGCCTA<br>CCGTGGCAATGACGGGTAACGGGGAATTAGGGTTCGATTCCGGAGAGGGAGCCTGAGAAACGGCTACCACATCTAAGGAAGGCAGCAGGCGCGCAA<br>ATTACCCAATCCTAATACAGGGAGGTAGTGACAAGAAATAACAATACAGGACCTTACGGTTTTGTAATTGGAATGAGTTAAGTATAAACCCCTTTACA<br>AGTATCAATTGGAGGGCAAGTCTGGTGCCAGCAGCCGCGGTAATTCCAGCTCCAATAGCGTATATTAAAGTTGTTGCAGTTAAAAAGCTCGTAGTTGG<br>ATTTCTGTTAATAATTTATATATAATATTACGGTATTTATATAATATTAACATAATTCATATTACTTTATTTTTAGAGTATATGAAATTTTACTTTGAGA<br>AAATTAGAGTGCTTAAAGCAGGCATATGCCTTGAATACTCCAGCATGGAATAATATTAAAGATTTTTATCTTTCTTATTGGTTCTAAGATAAAAATAA<br>TGATTAATAGGGACAGTTGGGGGCATTTGTATTTAACAGTCAGAGGTGAAATTCTTAGATTTGTTAAAGACAAACTAGTGCGAAAGCATTTGCCAAG<br>GATGTTTTCATTAATCAAGAACGAAAGTTAGGGGATCGAAGACGATC
PX496098	Cryptosporidium varanii LG60 Leopard gecko Chiang Mai Thailand	
PX496100	Cryptosporidium varanii LG12 Leopard gecko Chiang Mai Thailand	
PX496101	Cryptosporidium varanii LG40 Leopard gecko Chiang Mai Thailand	
PX496104	Cryptosporidium varanii LG3 Leopard gecko Chiang Mai Thailand	
PX496105	Cryptosporidium varanii LG17 Leopard gecko Chiang Mai Thailand	
PX496106	Cryptosporidium varanii LG98 Leopard gecko Chiang Mai Thailand	
PX496096	Cryptosporidium varanii LG61 Leopard gecko Chiang Mai Thailand	ATTATTTGGTGATTCATAATAACTTTACGGATCACAATTACTTGTGACATATCATTCAAGTTTCTGACCTATCAGCTTTAGACGGTAGGGTATTGGCCTA<br>CCGTGGCAATGACGGGTAACGGGGAATTAGGGTTCGATTCCGGAGAGGGAGCCTGAGAAACGGCTACCACATCTAAGGAAGGCAGCAGGCGCGCAA<br>ATTACCCAATCCTAATACAGGGAGGTAGTGACAAGAAATAACAATACAGGACCTTACGGTTTTGTAATTGGAATGAGTTAAGTATAAACCCCTTTACA<br>AGTATCAATTGGAGGGCAAGTCTGGTGCCAGCAGCCGCGGTAATTCCAGCTCCAATAGCGTATATTAAAGTTGTTGCAGTTAAAAAGCTCGTAGTTGG<br>ATTTCTGTTAATAATTTATATATAATATTACGGTATTTATATAATATTAACATAATTCATATTACTTTATTTTTAGAGTATATGAAATTTTACTTTGAGA<br>AAATTAGAGTGCTTAAAGCAGGCATATGCCTTGAATACTCCAGCATGGAATAATATTAAAGATTTTTATCTTTCTTATTGGTTCTAAGATAAAAATAA<br>TGATTAATAGGGACAGTTGGGGGCATTTGTATTTAACAGTCAGAGGTGAAATTCTTAGATTTGTTAAAGACAAACTAGTGCGAAAGCATTTGCCAAG<br>GATGTTTTCATTAATCAAGAACGAAAGTTAGGGGATCGAAGACGATC
PX496099	Cryptosporidium varanii LG79 Leopard gecko Chiang Mai Thailand	
PX496102	Cryptosporidium varanii LG11 Leopard gecko Chiang Mai Thailand	
PX496103	Cryptosporidium varanii LG31 Leopard gecko Chiang Mai Thailand	
PX496107	Cryptosporidium varanii LG66 Leopard gecko Chiang Mai Thailand	
