# Supplementary material for: Cryptosporidium varanii Infection in Captive Leopard Gecko (Eublepharis macularius) and Its Association with Wasting Syndrome in Thailand
Source: Animals (Basel). 2025 Dec 22;16(1):33. doi: 10.3390/ani16010033 (PMC12784683; doi:10.3390/ani16010033)
Supplement: Supplementary file 1 [file animals-16-00033-s001.zip › Supplement file Table S1.pdf]

Table S1 Body weight, Tail diameter, and BCS of leopard geckos, grouped by Cryptosporidium DNA positive and negative using nPCR-RFLP

Body Weight of Cryptosporidium positive geckos										
No.	Gecko ID	1	2	3	4	5	6	7	8	BW trend
1	1	64.3	60	53.7	54.1	54.6	56.7	63.6	65.3	
2	4	34.3	34.3	31.3	28.6	26.8	26	26.5	24.2	
3	5	66.5	68.3	67.2	65.7	65.8	66	68.9	70.6	
4	6	44.4	40.4	35.2	40.1	41.6	46.1	47.5	53.9	
5	9	69	66.9	63	63	71	77	83.2	88.9	
6	10	52.6	48	52.4	55.1	52.5	55.2	62.7	68.9	
7	22	66.9	57.7	64	59.8	61.6	66.8	68.7	72.8	
8	23	37.8	37.9	36.1	38.2	38.6	45.8	49.4	51.8	
9	25	39.1	38.7	40.1	39.8	41.7	45.4	49.1	50.6	
10	27	52.5	52.8	53.9	59.1	63.9	70.9	74.4	77.6	
11	28	65.2	64.4	62.7	61.8	65	69.2	69	70.1	
12	29	28.9	25	Death						
13	30	68.2	70.5	75	75	76.2	80.1	82.5	82.4	
14	31	66.3	67	66.3	65.3	64.6	71.5	68.7	78	
15	32	81	81.7	81.4	82	85.5	88.7	91.6	93.6	
16	33	48	46.1	49.6	50	52.2	53.8	55	57.5	
17	34	69.6	70.6	74.6	74.6	72.5	77.8	72.9	81.2	
18	35	59.7	55.1	62.2	61.9	67.2	70.2	79.4	76.3	

Tail diameter of Cryptosporidium positive geckos										
No.	Gecko ID	wk1	wk2	wk3	wk4	wk5	wk6	wk7	wk8	Tail ⌀ trend
1	1	12.4	13.3	12.5	13.3	13.3	14.8	14.1	16.1	
2	4	8.05	7.85	6.6	6	5.15	5.6	5	4.55	
3	5	14.3	16.2	16	16.3	15.2	15.5	18.2	20	
4	6	8.25	8.4	7.3	7.2	8.45	10.2	12.8	14.8	
5	9	12.2	13.9	13.6	12.2	14.6	15.1	17.8	18.6	
6	10	11.3	13.5	12.8	13	13.4	12.3	14.1	16.5	
7	22	13.1	13.3	13.2	13.1	14.2	18.4	17.2	16.9	
8	23	9.4	8.3	9.6	9.1	9.3	11.2	12.1	13.7	
9	25	12.4	12.6	11.2	11.2	12.1	13.7	12.2	14.9	
10	27	10.3	10.2	10.2	11.1	14	16.8	18.8	18.4	
11	28	10.4	9.3	11.1	11.2	10.5	13.5	14.6	14.7	
12	29	5.15	5.35	Death						
13	30	16.2	17	18	18	19.7	19.7	19.4	20.7	
14	31	14.4	15.4	14.3	14.2	15.1	16.1	16	19.8	
15	32	16.2	17.5	18.9	19	20.2	20.1	20.2	21.9	
16	33	12.4	11.2	12.9	12.9	13.4	14.8	14.7	16.2	
17	34	15.1	14.1	15.6	16.4	16.2	17.7	16.3	19.5	
18	35	11.3	13.7	13.2	14	18.5	17.4	18.1	17.8	

BCS of of Cryptosporidium positive geckos										
No.	Gecko ID	wk1	wk2	wk3	wk4	wk5	wk6	wk7	wk8	BCS trend
1	1	3	3	3	3	3	3	3	3	
2	4	2	2	2	1	1	1	1	1	
3	5	3	3	3	3	3	3	3	3	
4	6	3	1	1	1	2	2	3	3	
5	9	3	3	3	3	3	3	3	3	
6	10	3	3	3	3	3	3	3	3	
7	22	3	3	3	3	3	3	3	3	
8	23	2	2	2	2	2	2	2	3	
9	25	3	3	3	3	3	3	3	3	
10	27	3	2	3	3	3	3	3	3	
11	28	2	2	2	2	2	3	2	2	
12	29	1	1	Death						
13	30	5	5	5	5	5	5	5	5	
14	31	3	3	3	3	3	3	3	3	
15	32	3	3	3	3	3	3	3	3	
16	33	3	3	3	3	3	3	3	3	
17	34	3	3	3	3	3	3	3	3	
18	35	3	3	3	3	3	3	3	3	

Body Weight of Cryptosporidium negative geckos										
No.	Gecko ID	wk1	wk2	wk3	wk4	wk5	wk6	wk7	wk8	BW trend
1	2	58.4	58.1	57.6	56.2	52.9	53.8	55.8	57.8	
2	3	68.3	65.1	64.5	58.4	66.2	64	64	64.6	
3	7	56.2	53.4	51.7	48.9	43.5	48.2	50	54.7	
4	8	58.3	56.2	56.2	56.2	57.1	59.6	67.3	72.2	
5	11	95	94.8	95.5	93.7	95.7	101	98.6	110	
6	12	62.6	60.7	58.7	46.9	55.2	55	67.6	69.2	
7	13	66	62.3	59.9	59.7	60.3	65.9	66.2	70.7	
8	14	65	64.8	60.1	60.1	61.6	62.5	60.1	67.4	
9	15	64.8	60.6	60.3	60.1	62.5	68.7	71.3	75.5	
10	16	63.8	59.5	55.7	53.6	56.2	62.1	67.3	69	
11	17	58.3	60.4	62.7	57.8	66.6	76.3	78.2	80.2	
12	18	72	67.9	69.8	68.5	66.6	68.4	69.1	70.6	
13	19	61.2	58.4	58.9	56.9	58.4	62.9	64.9	69.1	
14	20	83.5	86.2	91.1	90.8	94.2	97	101	104	
15	21	50.2	48.9	46.7	52.1	58.3	65	63.8	68.8	
16	24	86.3	82.9	80	76.8	71.8	63.7	64.9	61.3	
17	26	32.2	31.4	33.5	32.8	35.5	37.9	39.7	40.6	

Tail diameter of Cryptosporidium negative geckos										
No.	Gecko ID	wk1	wk2	wk3	wk4	wk5	wk6	wk7	wk8	BW trend
1	2	18.3	18.8	17.1	17	15.5	15.1	15.8	16.9	
2	3	12.5	12.4	14.6	14.2	14.2	13.9	12.7	15.2	
3	7	16.6	18	16.2	14.5	13.4	13.6	13.2	16.2	
4	8	12.2	12.2	12.5	12.2	13	13.6	14.2	15.8	
5	11	18.2	18.6	17.9	17.6	18.3	20.9	20.5	21.2	
6	12	15.4	16.6	15.6	15.4	15.6	15.4	17.3	19.4	
7	13	14.2	13.2	13.8	13.2	17	17.8	17.5	16.3	
8	14	13.2	14.4	14.6	14.4	14.2	16.7	16.7	16.7	
9	15	16.4	15.7	15.9	15.4	17.7	18	18	18.2	
10	16	14.4	13.9	13.7	13.3	14.5	16.1	16.8	18.9	
11	17	13.1	13.4	14.9	14.3	17.7	18.6	17.2	20.7	
12	18	18.1	17.7	18.7	18.7	16.6	16.4	17.6	17.2	
13	19	15.4	14.1	14.9	14.9	16.2	17.8	17.9	18.5	
14	20	16.3	17.4	18.4	18.4	21.4	21.2	21	22	
15	21	9.35	8.1	9.15	10.2	13.2	15.6	16.5	16.1	
16	24	17.5	17.4	18.5	18.3	17.3	14.2	17.2	16.7	
17	26	9.05	10.2	11.3	11.5	13.8	13.9	15.7	15.7	

BCS of Cryptosporidium negative geckos										
No.	Gecko ID	wk1	wk2	wk3	wk4	wk5	wk6	wk7	wk8	BW trend
1	2	3	3	3	3	3	3	3	3	
2	3	3	3	3	3	3	3	3	3	
3	7	3	3	3	3	3	3	3	3	
4	8	3	3	3	3	3	3	3	3	
5	11	3	3	3	3	3	4	3	4	
6	12	3	3	3	3	3	3	3	3	
7	13	3	3	3	3	3	3	3	3	
8	14	3	3	3	3	3	3	3	3	
9	15	3	3	3	3	3	3	3	3	
10	16	3	3	3	3	3	3	3	3	
11	17	3	3	3	3	3	3	3	3	
12	18	4	3	3	3	3	3	3	3	
13	19	3	3	3	3	3	3	3	3	
14	20	3	3	3	3	3	3	3	3	
15	21	3	2	2	2	3	3	3	3	
16	24	5	5	5	5	4	4	3	3	
17	26	3	3	3	3	3	3	3	3	
