# Supplementary material for: Cryptosporidium varanii Infection in Captive Leopard Gecko (Eublepharis macularius) and Its Association with Wasting Syndrome in Thailand
Source: Animals (Basel). 2025 Dec 22;16(1):33. doi: 10.3390/ani16010033 (PMC12784683; doi:10.3390/ani16010033)
Supplement: Supplementary file 1 [file animals-16-00033-s001.zip › Supplement file Table S2.pdf]

**Table S2** Pictures of leopard geckos in each week during study period. Body weight, Tail diameter, and BCS and Cryptosporidium DNA positive and negative have been demonstrated.

nPCR Positive | nPCR Negative | nPCR (N/A) | Late Laying period

| General information | | | | | Data collection | | | | | | | | | | | | | | | | |
|---|---|---|---|---|---|---|---|---|---|---|---|---|---|---|---|---|---|---|---|---|---|
| | | | | | Study Period | 1 | | 2 | | 3 | | 4* | | 5* | | 6 | | 7 | | 8 | |
| Box | Number | Pictures | Sex | Age | Date & PCR result | 17/5/2021 | PCR | 3/6/2021 | PCR | 18/6/2021 | PCR | 3/7/2021 | PCR | 18/7/2021 | PCR | 4/8/2021 | PCR | 18/8/2021 | PCR | 4/9/2021 | PCR |
| 1 | 001 | | Female | 1 - 5 years | Weight (g) | 64.3 | | 60 | | 53.7 | | 54.1 | | 54.6 | | 56.7 | | 63.6 | | 65.3 | |
| | | | | | Tail diameter (mm.) | 12.35 | | 13.25 | | 12.45 | | 13.3 | | 13.3 | | 14.8 | | 14.1 | | 16.05 | |
| | | | | | BCS | 3 | | 3 | | 3 | | 3 | | 3 | | 3 | | 3 | | 3 | |
| 2 | 002 | | Female | 1 - 5 years | Weight (g.) | 58.4 | | 58.1 | | 57.6 | | 56.2 | | 52.9 | | 53.8 | | 55.8 | | 57.8 | |
| | | | | | Tail diameter (mm.) | 18.3 | | 18.8 | | 17.05 | | 17 | | 15.45 | | 15.1 | | 15.8 | | 16.9 | |
| | | | | | BCS | 3 | | 3 | | 3 | | 3 | | 3 | | 3 | | 3 | | 3 | |
| | 003 | | Female | 1 - 5 years | Weight (g.) | 68.3 | | 65.1 | | 64.5 | | 58.4 | | 66.2 | | 64 | | 64 | | 64.6 | |
| | | | | | Tail diameter (mm.) | 12.45 | | 12.4 | | 14.6 | | 14.2 | | 14.2 | | 13.85 | | 12.7 | | 15.2 | |
| | | | | | BCS | 3 | | 3 | | 3 | | 3 | | 3 | | 3 | | 3 | | 3 | |
| 3 | 004 | | Female | 1 - 5 years | Weight (g.) | 34.3 | | 34.3 | | 31.3 | | 28.6 | | 26.8 | | 26 | | 26.5 | | 24.2 | |
| | | | | | Tail diameter (mm.) | 8.05 | | 7.85 | | 6.6 | | 6 | | 5.15 | | 5.6 | | 5 | | 4.55 | |
| | | | | | BCS | 2 | | 2 | | 2 | | 1 | | 1 | | 1 | | 1 | | 1 | |
| | 005 | | Male | 1 - 5 years | Weight (g.) | 66.5 | | 68.3 | | 67.2 | | 65.7 | | 65.8 | | 66 | | 68.9 | | 70.6 | |
| | | | | | Tail diameter (mm.) | 14.25 | | 16.2 | | 16 | | 16.25 | | 15.15 | | 15.5 | | 18.15 | | 19.95 | |
| | | | | | BCS | 3 | | 3 | | 3 | | 3 | | 3 | | 3 | | 3 | | 3 | |
| | 006 | | Female | 1 - 5 years | Weight (g.) | 44.4 | | 40.4 | | 35.2 | | 40.1 | | 41.6 | | 46.1 | | 47.5 | | 53.9 | |
| | | | | | Tail diameter (mm.) | 8.25 | | 8.4 | | 7.3 | | 7.2 | | 8.45 | | 10.2 | | 12.8 | | 14.8 | |
| | | | | | BCS | 3 | | 1 | | 1 | | 1 | | 2 | | 2 | | 3 | | 3 | |
| 4 | 007 | | Female | 1 - 5 years | Weight (g.) | 56.2 | | 53.4 | | 51.7 | | 48.9 | | 43.5 | | 48.2 | | 50 | | 54.7 | |
| | | | | | Tail diameter (mm.) | 16.64 | | 18 | | 16.2 | | 14.45 | | 13.4 | | 13.6 | | 13.2 | | 16.2 | |
| | | | | | BCS | 3 | | 3 | | 3 | | 3 | | 3 | | 3 | | 3 | | 3 | |
| | 008 | | Female | 1 - 5 years | Weight (g.) | 58.3 | | 56.2 | | 56.2 | | 56.2 | | 57.1 | | 59.6 | | 67.3 | | 72.2 | |
| | | | | | Tail diameter (mm.) | 12.15 | | 12.15 | | 12.45 | | 12.2 | | 13 | | 13.55 | | 14.2 | | 15.8 | |
| | | | | | BCS | 3 | | 3 | | 3 | | 3 | | 3 | | 3 | | 3 | | 3 | |
| 5 | 009 | | Female | 1 - 5 years | Weight (g.) | 69 | | 66.9 | | 63 | | 63 | | 71 | | 77 | | 83.2 | | 88.9 | |
| | | | | | Tail diameter (mm.) | 12.2 | | 13.9 | | 13.55 | | 12.2 | | 14.6 | | 15.1 | | 17.8 | | 18.55 | |
| | | | | | BCS | 3 | | 3 | | 3 | | 3 | | 3 | | 3 | | 3 | | 3 | |
| | 010 | | Female | 1 - 5 years | Weight (g.) | 52.6 | | 48 | | 52.4 | | 55.1 | | 52.5 | | 55.2 | | 62.7 | | 68.9 | |
| | | | | | Tail diameter (mm.) | 11.25 | | 13.5 | | 12.8 | | 13 | | 13.35 | | 12.3 | | 14.05 | | 16.5 | |
| | | | | | BCS | 3 | | 3 | | 3 | | 3 | | 3 | | 3 | | 3 | | 3 | |
| 6 | 011 | | Female | 1 - 5 years | Weight (g.) | 95 | | 94.8 | | 95.5 | | 93.7 | | 95.7 | | 100.7 | | 98.6 | | 109.6 | |
| | | | | | Tail diameter (mm.) | 18.2 | | 18.6 | | 17.9 | | 17.6 | | 18.3 | | 20.9 | | 20.45 | | 21.15 | |
| | | | | | BCS | 3 | | 3 | | 3 | | 3 | | 3 | | 4 | | 3 | | 4 | |
| | 012 | | Female | 1 - 5 years | Weight (g.) | 62.6 | | 60.7 | | 58.7 | | 46.9 | | 55.2 | | 55 | | 67.6 | | 69.2 | |
| | | | | | Tail diameter (mm.) | 15.4 | | 16.6 | | 15.6 | | 15.4 | | 15.6 | | 15.35 | | 17.3 | | 19.4 | |
| | | | | | BCS | 3 | | 3 | | 3 | | 3 | | 3 | | 3 | | 3 | | 3 | |
| 7 | 013 | | Female | 1 - 5 years | Weight (g) | 66 | | 62.3 | | 59.9 | | 59.7 | | 60.3 | | 65.9 | | 66.2 | | 70.7 | |
| | | | | | Tail diameter (mm.) | 14.22 | | 13.15 | | 13.8 | | 13.2 | | 17 | | 17.8 | | 17.5 | | 16.25 | |
| | | | | | BCS | 3 | | 3 | | 3 | | 3 | | 3 | | 3 | | 3 | | 3 | |
| 8 | 014 | | Female | 1 - 5 years | Weight (g) | 65 | | 64.8 | | 60.1 | | 60.1 | | 61.6 | | 62.5 | | 60.1 | | 67.4 | |
| | | | | | Tail diameter (mm.) | 13.2 | | 14.35 | | 14.6 | | 14.4 | | 14.2 | | 16.65 | | 16.7 | | 16.65 | |
| | | | | | BCS | 3 | | 3 | | 3 | | 3 | | 3 | | 3 | | 3 | | 3 | |
| 9 | 015 | | Female | 1 - 5 years | Weight (g) | 64.8 | | 60.6 | | 60.3 | | 60.1 | | 62.5 | | 68.7 | | 71.3 | | 75.5 | |
| | | | | | Tail diameter (mm.) | 16.4 | | 15.7 | | 15.9 | | 15.35 | | 17.7 | | 17.95 | | 17.95 | | 18.2 | |
| | | | | | BCS | 3 | | 3 | | 3 | | 3 | | 3 | | 3 | | 3 | | 3 | |
| 10 | 016 | | Female | 1 - 5 years | Weight (g) | 63.8 | | 59.5 | | 55.7 | | 53.6 | | 56.2 | | 62.1 | | 67.3 | | 69 | |
| | | | | | Tail diameter (mm.) | 14.35 | | 13.85 | | 13.65 | | 13.25 | | 14.5 | | 16.1 | | 16.75 | | 18.9 | |
| | | | | | BCS | 3 | | 3 | | 3 | | 3 | | 3 | | 3 | | 3 | | 3 | |
| | 017 | | Female | 1 - 5 years | Weight (g) | 58.3 | | 60.4 | | 62.7 | | 57.8 | | 66.6 | | 76.3 | | 78.2 | | 80.2 | |
| | | | | | Tail diameter (mm.) | 13.1 | | 13.35 | | 14.85 | | 14.25 | | 17.7 | | 18.6 | | 17.2 | | 20.65 | |
| | | | | | BCS | 3 | | 3 | | 3 | | 3 | | 3 | | 3 | | 3 | | 3 | |
| | 018 | | Female | 1 - 5 years | Weight (g) | 72 | | 67.9 | | 69.8 | | 68.5 | | 66.6 | | 68.4 | | 69.1 | | 70.6 | |
| | | | | | Tail diameter (mm.) | 18.1 | | 17.7 | | 18.65 | | 18.65 | | 16.6 | | 16.4 | | 17.6 | | 17.2 | |
| | | | | | BCS | 4 | | 3 | | 3 | | 3 | | 3 | | 3 | | 3 | | 3 | |
| 11 | 019 | | Female | 1 - 5 years | Weight (g) | 61.2 | | 58.4 | | 58.9 | | 56.9 | | 58.4 | | 62.9 | | 64.9 | | 69.1 | |
| | | | | | Tail diameter (mm.) | 15.4 | | 14.05 | | 14.9 | | 14.85 | | 16.2 | | 17.8 | | 17.9 | | 18.5 | |
| | | | | | BCS | 3 | | 3 | | 3 | | 3 | | 3 | | 3 | | 3 | | 3 | |
| 12 | 020 | | Female | 1 - 5 years | Weight (g.) | 83.5 | | 86.2 | | 91.1 | | 90.8 | | 94.2 | | 97 | | 101.1 | | 103.7 | |
| | | | | | Tail diameter (mm.) | 16.25 | | 17.35 | | 18.35 | | 18.35 | | 21.4 | | 21.15 | | 20.95 | | 21.95 | |
| | | | | | BCS | 3 | | 3 | | 3 | | 3 | | 3 | | 3 | | 3 | | 3 | |
| | 021 | | Female | 1 - 5 years | Weight (g.) | 50.2 | | 48.9 | | 46.7 | | 52.1 | | 58.3 | | 65 | | 63.8 | | 68.8 | |
| | | | | | Tail diameter (mm.) | 9.35 | | 8.1 | | 9.15 | | 10.2 | | 13.2 | | 15.6 | | 16.5 | | 16.1 | |
| | | | | | BCS | 3 | | 2 | | 2 | | 2 | | 3 | | 3 | | 3 | | 3 | |
| 13 | 022 | | Female | 1 - 5 years | Weight (g) | 66.9 | | 57.7 | | 64 | | 59.8 | | 61.6 | | 66.8 | | 68.7 | | 72.8 | |
| | | | | | Tail diameter (mm.) | 13.1 | | 13.25 | | 13.2 | | 13.1 | | 14.15 | | 18.35 | | 17.15 | | 16.9 | |
| | | | | | BCS | 3 | | 3 | | 3 | | 3 | | 3 | | 3 | | 3 | | 3 | |
| 14 | 023 | | Female | < 1 year | Weight (g) | 37.8 | | 37.9 | | 36.1 | | 38.2 | | 38.6 | | 45.8 | | 49.4 | | 51.8 | |
| | | | | | Tail diameter (mm.) | 9.4 | | 8.3 | | 9.6 | | 9.1 | | 9.3 | | 11.2 | | 12.1 | | 13.7 | |
| | | | | | BCS | 2 | | 2 | | 2 | | 2 | | 2 | | 2 | | 2 | | 3 | |
| 15 | 024 | | Female | < 1 year | Weight (g) | 86.3 | | 82.9 | | 80 | | 76.8 | | 71.8 | | 63.7 | | 64.9 | | 61.3 | |
| | | | | | Tail diameter (mm.) | 17.5 | | 17.4 | | 18.5 | | 18.3 | | 17.3 | | 14.2 | | 17.15 | | 16.7 | |
| | | | | | BCS | 5 | | 5 | | 5 | | 5 | | 4 | | 4 | | 3 | | 3 | |
| 16 | 025 | | Female | < 1 year | Weight (g) | 39.1 | | 38.7 | | 40.1 | | 39.8 | | 41.7 | | 45.4 | | 49.1 | | 50.6 | |
| | | | | | Tail diameter (mm.) | 12.35 | | 12.55 | | 11.15 | | 11.15 | | 12.1 | | 13.65 | | 12.15 | | 14.9 | |
| | | | | | BCS | 3 | | 3 | | 3 | | 3 | | 3 | | 3 | | 3 | | 3 | |
| 17 | 026 | | Female | < 1 year | Weight (g) | 32.2 | | 31.4 | | 33.5 | | 32.8 | | 35.5 | | 37.9 | | 39.7 | | 40.6 | |
| | | | | | Tail diameter (mm.) | 9.05 | | 10.2 | | 11.3 | | 11.5 | | 13.8 | | 13.85 | | 15.7 | | 15.7 | |
| | | | | | BCS | 3 | | 3 | | 3 | | 3 | | 3 | | 3 | | 3 | | 3 | |
| 18 | 027 | | Female | 1 - 5 Years | Weight (g) | 52.5 | | 52.8 | | 53.9 | | 59.1 | | 63.9 | | 70.9 | | 74.4 | | 77.6 | |
| | | | | | Tail diameter (mm.) | 10.25 | | 10.2 | | 10.2 | | 11.05 | | 14 | | 16.8 | | 18.8 | | 18.4 | |
| | | | | | BCS | 3 | | 2 | | 3 | | 3 | | 3 | | 3 | | 3 | | 3 | |
| 19 | 028 | | Female | 1 - 5 Years | Weight (g) | 65.2 | | 64.4 | | 62.7 | | 61.8 | | 65 | | 69.2 | | 69 | | 70.1 | |
| | | | | | Tail diameter (mm.) | 10.35 | | 9.3 | | 11.05 | | 11.2 | | 10.5 | | 13.5 | | 14.6 | | 14.7 | |
| | | | | | BCS | 2 | | 2 | | 2 | | 2 | | 2 | | 3 | | 2 | | 2 | |
| | 029 | | Female | 1 - 5 Years | Weight (g) | 28.9 | | 25 | | | | | | | | | | | | | |
| | | | | | Tail diameter (mm.) | 5.15 | | 5.35 | | Death 17/6/2564 & Necropsy on 18/06/2564 | | | | | | | | | | | |
| | | | | | BCS | 1 | | 1 | | | | | | | | | | | | | |
| 20 | 030 | | Female | 1 - 5 Years | Weight (g) | 68.2 | | 70.5 | | 75 | | 75 | | 76.2 | | 80.1 | | 82.5 | | 82.4 | |
| | | | | | Tail diameter (mm.) | 16.2 | | 16.95 | | 17.95 | | 17.95 | | 19.7 | | 19.65 | | 19.4 | | 20.7 | |
| | | | | | BCS | 5 | | 5 | | 5 | | 5 | | 5 | | 5 | | 5 | | 5 | |
| 21 | 031 | | Female | 1 - 5 Years | Weight (g) | 66.3 | | 67 | | 66.3 | | 65.3 | | 64.6 | | 71.5 | | 68.7 | | 78 | |
| | | | | | Tail diameter (mm.) | 14.4 | | 15.4 | | 14.25 | | 14.15 | | 15.1 | | 16.1 | | 16 | | 19.8 | |
| | | | | | BCS | 3 | | 3 | | 3 | | 3 | | 3 | | 3 | | 3 | | 3 | |
| | 032 | | Male | 1 - 5 Years | Weight (g) | 81 | | 81.7 | | 81.4 | | 82 | | 85.5 | | 88.7 | | 91.6 | | 93.6 | |
| | | | | | Tail diameter (mm.) | 16.15 | | 17.5 | | 18.85 | | 18.95 | | 20.15 | | 20.1 | | 20.2 | | 21.9 | |
| | | | | | BCS | 3 | | 3 | | 3 | | 3 | | 3 | | 3 | | 3 | | 3 | |
| 22 | 033 | | Female | 1 - 5 Years | Weight (g) | 48 | | 46.1 | | 49.6 | | 50 | | 52.2 | | 53.8 | | 55 | | 57.5 | |
| | | | | | Tail diameter (mm.) | 12.4 | | 11.15 | | 12.9 | | 12.9 | | 13.4 | | 14.8 | | 14.7 | | 16.2 | |
| | | | | | BCS | 3 | | 3 | | 3 | | 3 | | 3 | | 3 | | 3 | | 3 | |
| 23 | 034 | | Female | 1 - 5 Years | Weight (g) | 69.6 | | 70.6 | | 74.6 | | 74.6 | | 72.5 | | 77.8 | | 72.9 | | 81.2 | |
| | | | | | Tail diameter (mm.) | 15.1 | | 14.1 | | 15.6 | | 16.35 | | 16.2 | | 17.65 | | 16.3 | | 19.5 | |
| | | | | | BCS | 3 | | 3 | | 3 | | 3 | | 3 | | 3 | | 3 | | 3 | |
| | 035 | | Male | 1 - 5 Years | Weight (g) | 59.7 | | 55.1 | | 62.2 | | 61.9 | | 67.2 | | 70.2 | | 79.4 | | 76.3 | |
| | | | | | Tail diameter (mm.) | 11.25 | | 13.7 | | 13.2 | | 14 | | 18.45 | | 17.4 | | 18.05 | | 17.8 | |
| | | | | | BCS | 3 | | 3 | | 3 | | 3 | | 3 | | 3 | | 3 | | 1 - 5 Years | |

12 positive cages/23 cages (52.17%)
18 positive geckos/35 cages (51.43%)

1
1
